# Targeting MET and mTOR Synergistically Overcomes Adaptive Resistance in Glioblastoma

**DOI:** 10.3390/ijms27177780

**Published:** 2026-08-30

**Authors:** Yunzhan Li, Hanif Khan, Seyma Demirsoy, Muhammad Younis, Guilan Shi, Hannah Valensi, William Bernhardt, Jeongwu Lee, Mitchell Machtay, Dawit Aregawi, Michael Glantz, Pierre Giglio, Shengyu Yang, Todd Schell, Vonn Walter, Yasin Uzun, Inan Olmez

**Affiliations:** 1 Institute of Translational Medicine, China Pharmaceutical University, Nanjing 210009, China; 2 Departments of Radiation Oncology, Penn State University, Hershey, PA 17033, USA; 3 Departments of Neurosurgery, Penn State University, Hershey, PA 17033, USA; 4 Department of Neurosurgery, Virginia Commonwealth University, Richmond, VA 23298, USA; 5 Departments of Pediatrics, Penn State University, Hershey, PA 17033, USA; 6 Department of Stem Cell Biology and Regenerative Medicine, Lerner Research Institute, Cleveland Clinic, Cleveland, OH 44195, USA; 7 Department of Neurology, Ohio State University, Columbus, OH 43210, USA; 8 Departments of Cellular and Molecular Physiology, Penn State University, Hershey, PA 17033, USA; 9 Departments of Cell and Biological Systems, Penn State University, Hershey, PA 17033, USA; 10 Departments of Public Health Sciences, Penn State University, Hershey, PA 17033, USA; 11 Massey Comprehensive Cancer Center, Virginia Commonwealth University, 401 College St, Richmond, VA 23298, USA

**Keywords:** MET, BNIP3, mTOR, Glioblastoma, crizotinib, everolimus

## Abstract

Glioblastoma (GBM) is the most aggressive primary brain tumor with limited therapeutic options and extremely poor prognosis. Aberrant activation of the receptor tyrosine kinase MET drives tumor progression, therapeutic resistance, and reduced survival, particularly in the mesenchymal GBM subtype. Given its crucial role in GBM recurrence and progression, we investigated the mechanisms of resistance to MET inhibition using patient-derived glioma-initiating cells (GICs) and orthotopic xenograft mouse models. GICs were treated with the MET inhibitor crizotinib to elucidate the mechanism of adaptive resistance. Prolonged MET inhibition induced a senescent-like phenotype in GICs, associated with downregulation of BNIP3, a mitochondrial protein regulating mitophagy. We showed that BNIP3 downregulation led to activation of mTOR signaling, promoting cellular survival and adaptive resistance. Combining crizotinib with the mTOR inhibitor everolimus effectively suppressed mTOR activity, reduced cell viability, and induced mitochondrial alterations, apoptosis, and necroptosis. In orthotopic GBM xenograft models, combined MET and mTOR inhibition significantly prolonged survival compared with single-agent treatments. Notably, sequential treatment—crizotinib followed by everolimus—further enhanced therapeutic efficacy. These effects were achieved without significant weight loss, supporting tolerability of the treatment regimen. Our findings identify the BNIP3-mTOR axis as a critical mediator of resistance to MET inhibition and demonstrate that combined inhibition of MET and mTOR exhibits significant synergy against GBM.

## 1. Introduction

Glioblastoma (GBM) is the most common and most lethal primary brain malignancy [1]. It is characterized by extensive infiltration into surrounding brain parenchyma, rapid proliferation, and profound resistance to conventional therapies. Despite multimodal treatment consisting of maximal surgical resection followed by radiotherapy and temozolomide chemotherapy, patient outcomes remain dismal, with a median survival of only 15–18 months following diagnosis [2], underscoring the urgent need for new therapies. Receptor tyrosine kinase (RTK) signaling pathways are dysregulated in 80–90% of GBMs through genetic and non-genetic modifications [3,4]. These signaling abnormalities play a central role in tumor growth, invasion, and therapeutic resistance, and have therefore positioned RTKs as attractive targets for GBM therapy. However, despite extensive clinical evaluation of RTK inhibitors, durable clinical benefit has remained elusive.

Among RTKs, the hepatocyte growth factor (HGF)/MET signaling axis has emerged as a particularly compelling therapeutic target in GBM. Activation of MET triggers multiple downstream pathways, including PI3K/AKT, RAS/MAPK, and STAT3, which collectively promote tumor cell survival, proliferation, invasion, and the maintenance of glioma stem-like populations [5,6,7,8]. Aberrant MET activation is frequently observed in GBM and is particularly enriched at invasive tumor margins, where it contributes to the highly infiltrative and aggressive phenotype of this tumor [9]. Moreover, MET signaling has been directly implicated in therapeutic resistance by enhancing DNA damage repair following irradiation and promoting tumor cell survival under hypoxic stress, thereby diminishing the efficacy of standard treatments such as radiotherapy and temozolomide [10]. These properties have driven substantial interest in MET as a therapeutic target in GBM.

Accordingly, several MET inhibitors, including crizotinib and cabozantinib, have been evaluated in preclinical GBM models and early-phase clinical trials, with mixed therapeutic outcomes [9,11,12,13]. While MET-directed therapies can elicit initial anti-tumor responses, their clinical efficacy as monotherapies has been limited and transient, largely due to the rapid development of resistance [14,15]. Resistance to MET-targeted monotherapy arises through adaptive signaling rewiring and engagement of compensatory survival pathways that sustain downstream oncogenic signaling despite effective MET inhibition [16,17,18]. However, the molecular mechanisms governing this adaptive resistance remain incompletely understood.

In this study, we identify a previously unrecognized mechanism underlying resistance to MET inhibition in GBM. We demonstrate that MET inhibitor crizotinib exhibits anti-tumor activity, at least in part, through suppression of BNIP3, a mitochondrial protein that regulates mitophagy and cell death. We further show that BNIP3 downregulation results in activation of mTOR signaling, thereby promoting adaptive survival responses following MET inhibition. Importantly, pharmacologic inhibition of mTOR with everolimus markedly enhances the anti-tumor efficacy of crizotinib both in vitro and in vivo. Together, our findings uncover a BNIP3–mTOR signaling axis as a critical determinant of MET inhibitor response and provide a strong rationale for combinatorial targeting of MET and mTOR as a therapeutic strategy in GBM.

## 2. Results

### 2.1. MET Expression Is Elevated in GBM and Correlates with Poor Patient Survival

**Figure 1:**
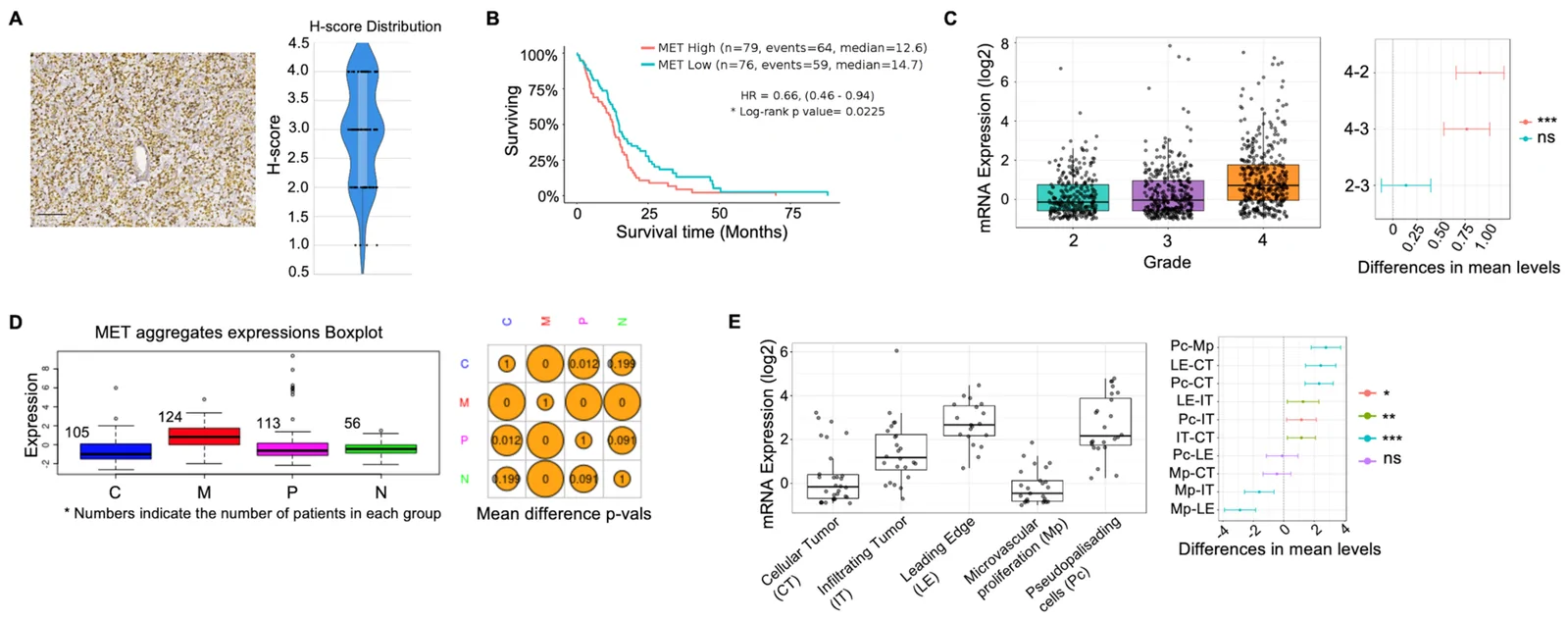
**MET expression in glioblastoma associates with tumor aggressiveness and poor survival.** (**A**) Representative immunohistochemical staining of MET in human GBM tissue (**left**) and quantification of MET expression using H-score analysis (**right**), demonstrating high MET expression across GBM samples. All samples included in this analysis were IDH-wildtype. n = 28 GBM samples (Ivy GBM Atlas Project). Scale bar, 100 μm. (**B**) Kaplan–Meier survival analysis of GBM patients (IDH wildtype) from the TCGA database stratified by MET expression levels, showing significantly shorter survival in patients with high MET expression. Patients were grouped into MET High and MET Low groups using the median MET expression level as the cutoff. Cox regression analysis was performed with MET High as the reference group; the hazard ratio (HR) and log-rank *p* value are indicated. (**C**) MET mRNA expression across glioma grades (2–4) in the TCGA data, showing a progressive increase in MET expression with advancing tumor grade. The right panel shows pairwise comparisons of mean expression differences between grades. (**D**) MET mRNA expression across molecular GBM subtypes—classical (C), mesenchymal (M), proneural (P), and neural (N)—demonstrating significant enrichment of MET expression in the mesenchymal subtype. The neural subtype is considered to largely reflect normal brain tissue contamination. Numbers indicate patient counts per group; the right panel shows pairwise mean difference *p* values. (**E**) MET mRNA expression (Ivy_GAP) across distinct anatomic tumor regions, including cellular tumor (CT), infiltrating tumor (IT), leading edge (LE), microvascular proliferation (Mp), and pseudopalisading cells (Pc), showing preferential enrichment of MET expression in invasive and aggressive tumor compartments. The right panel depicts pairwise comparisons of mean expression differences.

We assessed MET protein expression levels in GBM patient samples and observed high MET expression across GBM samples (Figure 1A). All samples included in this analysis were IDH-wildtype. For clinical relevance, analysis of IDH wild-type GBM data from The Cancer Genome Atlas (TCGA) revealed that higher MET expression was significantly associated with shorter overall survival (Figure 1B). Consistent with these findings, TCGA analysis showed that MET expression progressively increased with glioma grade. However, this analysis was not stratified by IDH status. An independent analysis stratified by IDH status demonstrated significantly higher MET expression in IDH-wildtype compared with IDH-mutant gliomas, supporting the association between MET expression and more aggressive molecular features (Figure 1C and Figure S1). GBM has been classified into four molecular subtypes—classical, proneural, neural, and mesenchymal—based on gene expression profiling [19,20]. In epithelial malignancies, epithelial–mesenchymal transition (EMT) enables cancer cells to acquire mesenchymal features, associated with therapeutic resistance, enhanced invasiveness, and metastatic potential [21]. Similarly, in GBM, acquisition of a mesenchymal phenotype is linked to increased tumor aggressiveness and resistance to therapy, underscoring the need for therapeutic strategies targeting this phenotype. TCGA analysis revealed significantly higher MET mRNA expression in the mesenchymal subtype compared with other GBM subtypes (Figure 1D). Consistently, MET expression was enriched in infiltrating tumor regions, the leading edge, and pseudopalisading cells, further supporting its association with aggressive tumor features (Figure 1E).

### 2.2. Prolonged MET Inhibition Induces Senescence and Alters Transcriptional Programs in GICs

**Figure 2:**
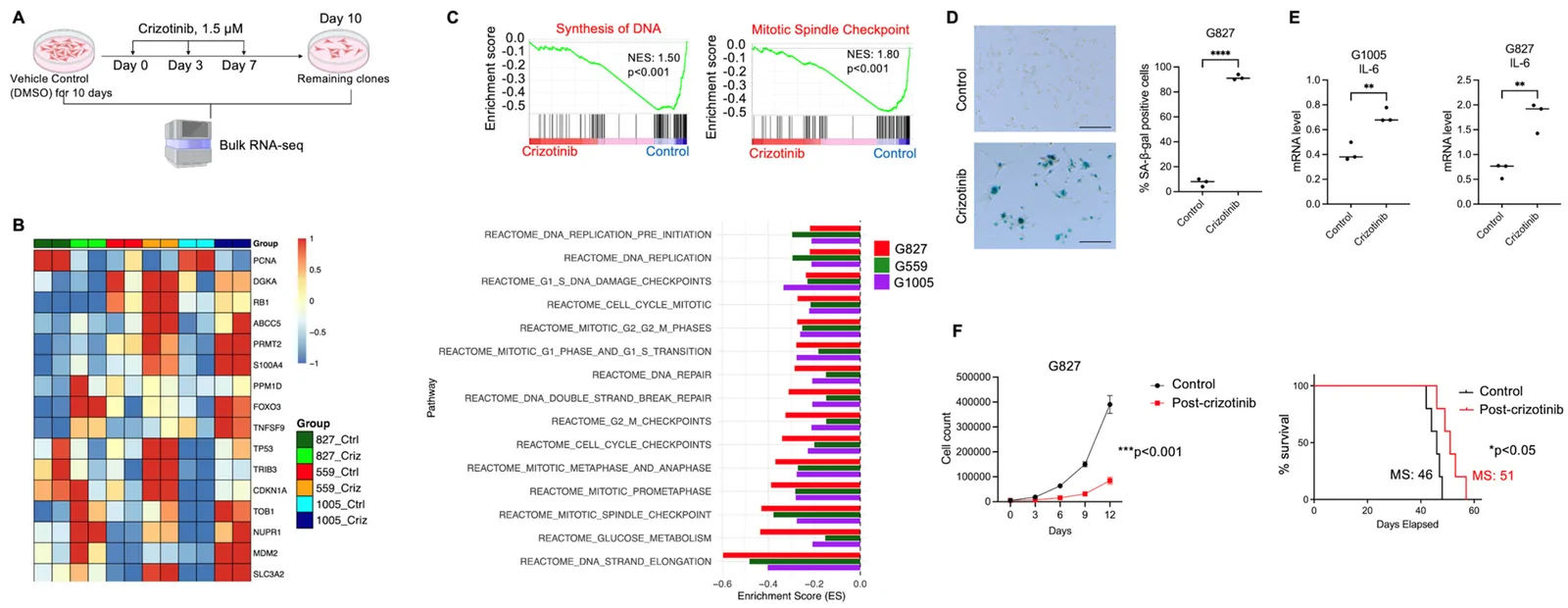
**MET inhibition induces a senescence-like state and suppresses proliferative programs in GICs.** (**A**) Experimental design: GICs were treated with crizotinib for three cycles (3 days per cycle), whereas control cells received DMSO vehicle treatment. Both groups were maintained in culture for 10 days, and surviving cells were collected for bulk RNA sequencing. (**B**) Heatmap of differentially expressed genes showing upregulation of p53 signaling and senescence-associated genes. (**C**) GSEA and Reactome pathway analysis demonstrating significant downregulation of DNA synthesis, mitosis, and cell cycle progression in crizotinib-treated cells. Normalized enrichment scores (NES) and *p* values are indicated. (**D**) Representative images of senescence-associated β-galactosidase (SA-β-gal) staining in control and crizotinib-treated GICs, showing increased senescent cells following MET inhibition. Crizotinib (0.75 µM). Scale bar, 200 µm. Data are shown as mean ± SEM. (**** *p* < 0.0001; two-tailed *t*-test). (**E**) Quantitative RT–PCR analysis of IL-6 mRNA expression in GICs following crizotinib (0.75 µM) treatment, indicating induction of a senescence-associated secretory phenotype (SASP). Data are shown as mean ± SEM. (* *p* < 0.05, ** *p* < 0.01, *** *p* < 0.001; two-tailed *t*-test). (**F**) Left panel: Cell proliferation curves showing reduced growth of GICs following prolonged crizotinib treatment (two-tailed *t*-test). Right panel: Kaplan–Meier survival analysis demonstrating extended survival in mice implanted with crizotinib-pretreated GICs compared with controls. Median survival (MS) values and *p* values are indicated. Data are presented as mean ± SEM from three independent biological replicates.

To investigate the transcriptional response to MET inhibition, glioma-initiating cells (GICs) were treated with the MET inhibitor crizotinib for three cycles (3 days per cycle). On day 10, surviving clones were collected for bulk RNA sequencing together with untreated control cells (Figure 2A and Figure S2A). GICs were exposed to high-dose crizotinib (1.5 µM) for three treatment cycles to eliminate sensitive populations and enrich surviving resistant clones for bulk RNA sequencing. RNA-seq analysis revealed significant upregulation of genes associated with p53 signaling and cellular senescence in crizotinib-treated GICs (Figure 2B). GSEA and pathway analysis showed downregulation of DNA synthesis, cell cycle progression, and mitosis in treated cells compared with controls (Figure 2C and Figure S2B). Senescence-associated changes were validated by β-Galactosidase staining, which showed a marked increase in β-galactosidase-positive cells following prolonged crizotinib treatment (Figure 2D and Figure S2C). Consistently, mRNA levels of senescence-associated cytokines, including IL-6, CXCL10, and CXCL8, were increased (Figure 2E and Figure S2D). GSEA further revealed enrichment of epigenetic regulation pathways, including DNA and histone lysine methylation, consistent with chromatin remodeling associated with crizotinib-induced senescence-like changes (Figure S2E,F). Collectively, these results demonstrate that prolonged MET inhibition induces a senescence-like program in GICs while concomitantly suppressing proliferation- and metabolism-associated pathways. Finally, we assessed the stability of this senescence-like state following MET inhibition. Upon crizotinib withdrawal, GICs initially showed reduced proliferation consistent with a senescence-like phenotype, but gradually resumed growth, although at a slower rate than untreated controls. Similarly, in an orthotopic brain tumor model, crizotinib-treated GICs showed prolonged survival compared with controls, although the difference was modest, suggesting that the induced senescence-like state is not permanent (Figure 2F).

### 2.3. MET Inhibition Suppresses BNIP3 and Alters Mitochondrial Homeostasis in GICs

**Figure 3:**
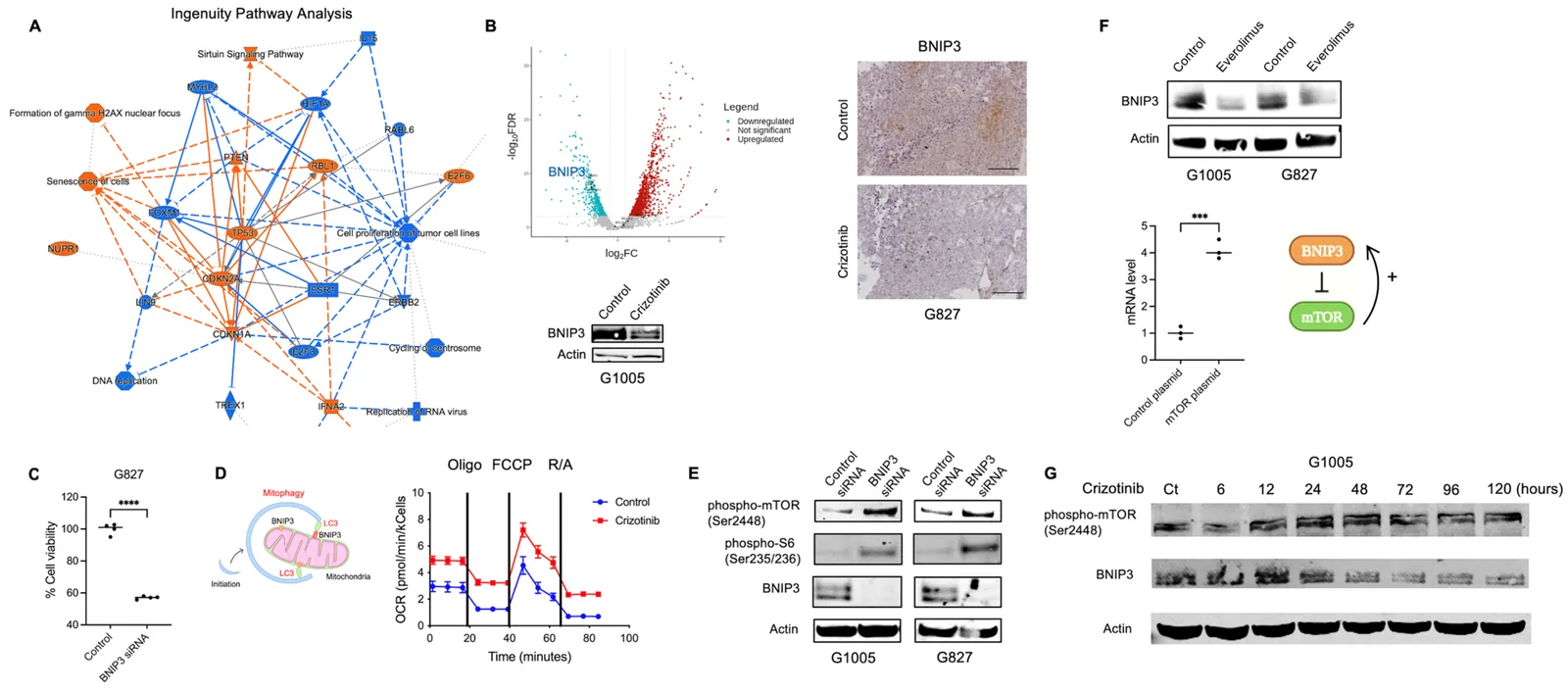
**BNIP3 downregulation links MET inhibition to altered mitochondrial homeostasis and mTOR activation.** (**A**) Ingenuity Pathway Analysis (IPA) network generated from bulk RNA-seq data of crizotinib-treated GICs. (**B**) Left: Volcano plot showing differentially expressed genes following crizotinib treatment, with BNIP3 significantly downregulated. Right: Representative immunohistochemical staining of BNIP3 in orthotopic GBM mouse xenografts, comparing control and crizotinib-treated groups, demonstrating reduced BNIP3 expression upon MET inhibition. Scale bar, 50 µm. Lower panel: Immunoblot analysis confirming BNIP3 downregulation following crizotinib treatment in GICs. (**C**) Cell viability assay following BNIP3 silencing by siRNA, showing a significant reduction in cell viability compared with control siRNA. Data are shown as mean ± SEM (**** *p* < 0.0001; two-tailed *t*-test). (**D**) Left panel: Schematic illustrating the role of BNIP3 in mitophagy through LC3-mediated mitochondrial sequestration. Right panel: Oxygen consumption rate (OCR) and the maximal OCR were measured using a Seahorse XF 24 Flux Analyzer in GICs subjected to a mitochondrial stress test. *** *p* < 0.001; one-way ANOVA with post-hoc Tukey analysis. Values are mean ± SEM of triplicates. Crizotinib dose was 0.75 µM. (**E**) Immunoblot analysis demonstrating activation of mTOR signaling upon BNIP3 loss. (**F**) Upper panel: Immunoblot showing decreased BNIP3 protein levels following mTOR inhibition with everolimus. Lower panel: Quantitative RT–PCR analysis showing increased BNIP3 expression following mTOR overexpression, indicating positive regulation of BNIP3 by mTOR signaling. Data are shown as mean ± SEM. (*** *p* < 0.001; two-tailed *t*-test). The schematic depicts a reciprocal negative feedback loop in which mTOR positively regulates BNIP3 expression, whereas BNIP3 negatively regulates mTOR signaling. (**G**) Time-course immunoblot analysis of GICs treated with crizotinib (0.75 µM) at the indicated time points, demonstrating progressive mTOR activation concomitant with BNIP3 downregulation during prolonged MET inhibition. Data are presented as mean ± SEM from three independent biological replicates.

To further elucidate crizotinib-induced transcriptional changes, pathway analysis using Ingenuity Pathway Analysis (IPA) confirmed activation of p53- and senescence-associated pathways and suppression of cell cycle programs, while also revealing downregulation of HIF1α-mediated hypoxia signaling upon crizotinib treatment (Figure 3A). *BNIP3*, a canonical HIF1α target gene involved in hypoxic stress adaptation and mitochondrial homeostasis, was prioritized among the significantly downregulated genes associated with HIF1α-mediated hypoxia signaling based on its established role in mitochondrial quality control and stress adaptation through regulation of mitophagy (Figure 3B). A comprehensive list of genes significantly downregulated following crizotinib treatment is provided in Supplementary Table S1. Analysis of TCGA GBM data revealed a significant correlation between BNIP3 expression and other HIF1α-regulated genes, supporting its association with hypoxia signaling (Figure S3A). Consistent with these transcriptomic findings, BNIP3 protein levels were markedly decreased in crizotinib-treated tumors, as confirmed by immunoblotting and IHC (Figure 3B). BNIP3 expression was detected at high levels in GBM patient samples (Figure S3B), suggesting its potential involvement in GBM pathophysiology.

**Figure 4:**
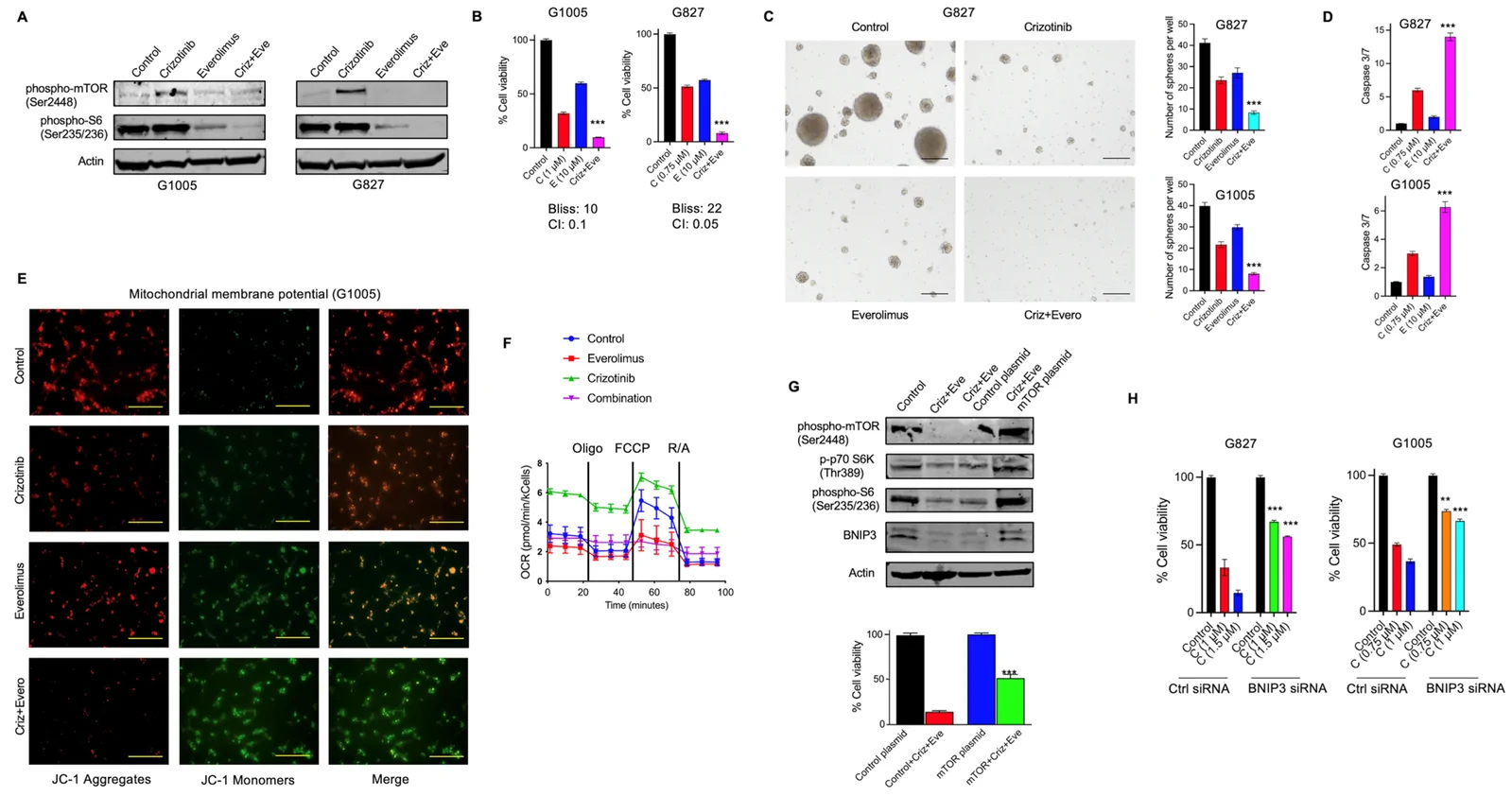
**Combined MET and mTOR inhibition exhibits synergy against GICs.** (**A**) Immunoblot analysis of mTOR signaling in GICs treated with crizotinib, everolimus, and their combination (Criz+Eve). (**B**) The combination of crizotinib (C) and everolimus (E) is synergistic against GICs (each treatment condition received either DMSO or the indicated drug) (*** *p* < 0.001; one-way ANOVA with post-hoc Tukey analysis). Synergy was evaluated using Bliss independence and combination index (CI) analysis, demonstrating strong synergistic effects. (**C**) 100 GICs were cultured in 24-well plates over one week to assess self-renewal capacity under vehicle, crizotinib (0.75 µM), everolimus (5 µM), or combination treatment (*** *p* < 0.0001; one-way ANOVA with post-hoc Tukey analysis). Scale bars, 100 µm. (**D**) Caspase-3/7 activity following three days of combination treatment, showing increased apoptotic activity (*** *p* < 0.0001; one-way ANOVA with post-hoc Tukey analysis). (**E**) Assessment of mitochondrial membrane potential in G1005 cells using JC-1 staining. Red fluorescence indicates JC-1 aggregates (intact mitochondrial membrane potential), whereas green fluorescence indicates JC-1 monomers (depolarized mitochondria). Scale bars, 200 µm. (**F**) Oxygen consumption rate (OCR) analysis measured by Seahorse assay in cells treated with everolimus, crizotinib, or the combination, showing reversal of crizotinib-induced mitochondrial respiration by combination treatment. (**G**) Immunoblot analysis of mTOR signaling and BNIP3 in GICs transfected with control or mTOR plasmid and treated with Criz+Eve. Bottom panel: Quantification of cell viability under the indicated conditions (*** *p* < 0.0001; two-way ANOVA). (**H**) Cell viability of GICs transfected with control or BNIP3 siRNA and treated with crizotinib, showing partial rescue of drug sensitivity upon BNIP3 depletion (** *p* < 0.01, *** *p* < 0.0001; two-way ANOVA). Data are presented as mean ± SEM from three independent biological replicates.

Functional silencing of BNIP3 significantly reduced cell viability and sphere-forming capacity in GICs (Figure 3C and Figure S3C), supporting its role in GIC survival. IPA pathway analysis revealed enrichment of a mitochondrial dysfunction-associated signature following prolonged crizotinib treatment (Figure S3D), suggesting that BNIP3 suppression, a key regulator of mitophagy and mitochondrial homeostasis, may contribute to altered mitochondrial homeostasis. Given that crizotinib downregulates BNIP3, we next investigated its impact on mitochondrial function in GICs. Consistently, Seahorse analysis demonstrated that crizotinib-treated GICs exhibited increased oxygen consumption rate (OCR), indicative of altered mitochondrial activity (Figure 3D). Because the increase in mitochondrial activity observed with crizotinib treatment could not be solely attributed to BNIP3 downregulation, we investigated potential adaptive resistance mechanisms contributing to this effect. BNIP3 has been reported to interact with Rheb and suppress mTOR signaling [22] (Figure S3E); therefore, we hypothesized that crizotinib-induced BNIP3 downregulation might enhance mTOR activity. To test this, we assessed mTOR signaling following BNIP3 silencing by siRNA. BNIP3 silencing increased phospho-mTOR and phospho-S6 levels, indicating activation of the mTOR pathway (Figure 3E). To examine whether mTOR regulates BNIP3, we showed that pharmacologic inhibition of mTOR with everolimus reduced BNIP3 expression, whereas mTOR overexpression using an mTOR plasmid significantly elevated BNIP3 levels (Figure 3F, see also Figure 4G), suggesting a bidirectional regulatory relationship between BNIP3 and mTOR signaling in GICs. Moreover, time-course immunoblot analysis following crizotinib treatment revealed an inverse correlation between BNIP3 expression and mTOR activity, with prolonged treatment leading to progressive BNIP3 downregulation concomitant with increased mTOR activation (Figure 3G and Figure S3F). MET silencing using siRNA similarly induced mTOR activation (Figure S3G), further highlighting the interplay between BNIP3, mTOR signaling, and MET in compensatory resistance mechanisms.

### 2.4. Combined MET and mTOR Inhibition Exhibits Synergy Against GICs

Given the activation of mTOR signaling following MET inhibition, we next evaluated whether pharmacologic mTOR inhibition could overcome this adaptive response and enhance the antitumor efficacy of crizotinib. The mTOR inhibitor everolimus effectively suppressed crizotinib-induced mTOR activation and downstream signaling (Figure 4A and Figure S4A). Combined MET and mTOR inhibition using crizotinib and everolimus led to a significantly greater reduction in GIC viability and sphere-forming capacity compared with either treatment alone, indicating that mTOR inhibition sensitizes GICs to crizotinib (Figure 4B,C and Figure S4B,C). Everolimus was used at 5 µM based on our previous studies demonstrating effective suppression of GIC self-renewal and inhibition of mTOR pathway activity. Consistent with these findings, the combination of crizotinib and everolimus significantly activated apoptotic signaling, whereas single-agent treatments resulted in minimal apoptotic activity (Figure 4D).

Given the potential role of mTOR signaling to crizotinib-induced mitochondrial activation, we next assessed the effects of combined crizotinib and everolimus treatment on mitochondrial function. JC-1 staining revealed that the combination treatment increased the proportion of monomeric JC-1, indicating mitochondrial depolarization, while significantly reducing JC-1 aggregate formation (Figure 4E and Figure S4D). Consistently, Seahorse OCR analysis showed that the combination treatment reversed the crizotinib-induced increase in mitochondrial respiration (Figure 4F), suggesting that mTOR inhibition attenuates adaptive mitochondrial activation. Moreover, as shown in Figure S4E, the combination therapy further increased cellular senescence, as evidenced by increased β-Galactosidase activity, supporting the therapeutic efficacy of this approach. To directly assess the role of mTOR in mediating resistance to crizotinib, mTOR overexpression via plasmid largely restored BNIP3 levels and rescued cell viability in cells treated with the combination therapy, confirming that mTOR signaling regulates BNIP3 and modulates GIC sensitivity to crizotinib (Figure 4G and Figure S4F). Finally, since crizotinib reduces BNIP3 expression in GICs, we investigated whether BNIP3 depletion influences cellular response to crizotinib using BNIP3-specific siRNA. BNIP3 knockdown partially rescued crizotinib-induced cytotoxicity and decreased cellular sensitivity to crizotinib, suggesting that BNIP3 downregulation contributes to the anti-tumor effects of crizotinib (Figure 4H).

### 2.5. Combined MET and mTOR Inhibition Induces Necroptosis in Crizotinib-Treated GICs

**Figure 5:**
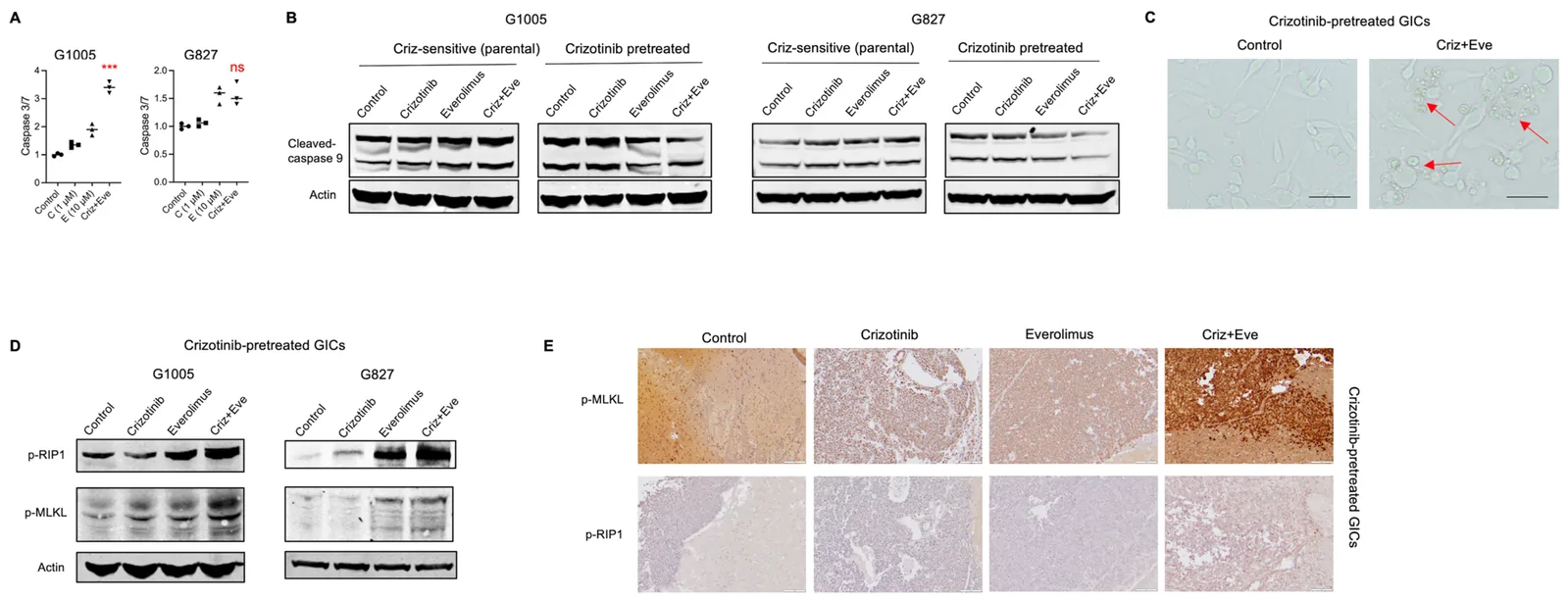
**Combined crizotinib and everolimus treatment induces necroptotic cell death in crizotinib-pretreated GICs.** (**A**) Caspase-3/7 activity in crizotinib-sensitive (parental) and crizotinib-pretreated GICs treated with crizotinib (C), everolimus (E), or the combination (Criz+Eve). While a significant increase in caspase-3/7 activity was observed in parental cells, minimal caspase-3/7 activation was detected in crizotinib-pre-treated cells (*** *p* < 0.0001; one-way ANOVA, ns = non-significant). (**B**) Immunoblot analysis of cleaved caspase-9 in parental crizotinib-sensitive GICs and crizotinib-pretreated GICs following treatment with crizotinib, everolimus, or their combination. (**C**) Representative phase-contrast images showing morphological changes associated with cell death following Criz+Eve treatment compared with control. Arrows indicate rounded and detached cells. Scale bar, 200 µm. (**D**) Immunoblot analysis in crizotinib-pretreated GICs following the indicated treatments, indicating activation of necroptotic signaling upon combination therapy. (**E**) Immunohistochemical staining of orthotopic GBM mouse xenografts from control, crizotinib, everolimus, or Criz+Eve groups, demonstrating enhanced necroptosis in tumors receiving combination therapy. Scale bar, 50 µm.

To further elucidate the cell death mechanisms induced by the combination of crizotinib and everolimus, we also assessed apoptosis in GICs pre-treated with crizotinib. While the combination treatment induced significant apoptotic activity in untreated parental cells (Figure 4D), apoptosis levels were minimal in cells pre-treated with crizotinib for three cycles before everolimus was added (Figure 5A,B). Because RIPK3 expression is required for canonical necroptotic signaling, we confirmed RIPK3 expression in the GIC models used in this study by qRT-PCR (Figure S5). Morphologically, these cells exhibited necrotic features, suggesting a transition toward a caspase-independent cell death phenotype with necroptotic features (Figure 5C). Consistent with this observation, combined treatment induced activation of necroptosis-associated signaling, including increased phosphorylation of RIP1 and MLKL in GICs and tumor xenografts (Figure 5D,E). Collectively, these findings indicate that crizotinib-pretreated cells undergo a caspase-independent cell death response with molecular features consistent with necroptosis following combined crizotinib and everolimus treatment.

### 2.6. Combined MET and mTOR Inhibition Is Effective in Aggressive Orthotopic GIC Models In Vivo

**Figure 6:**
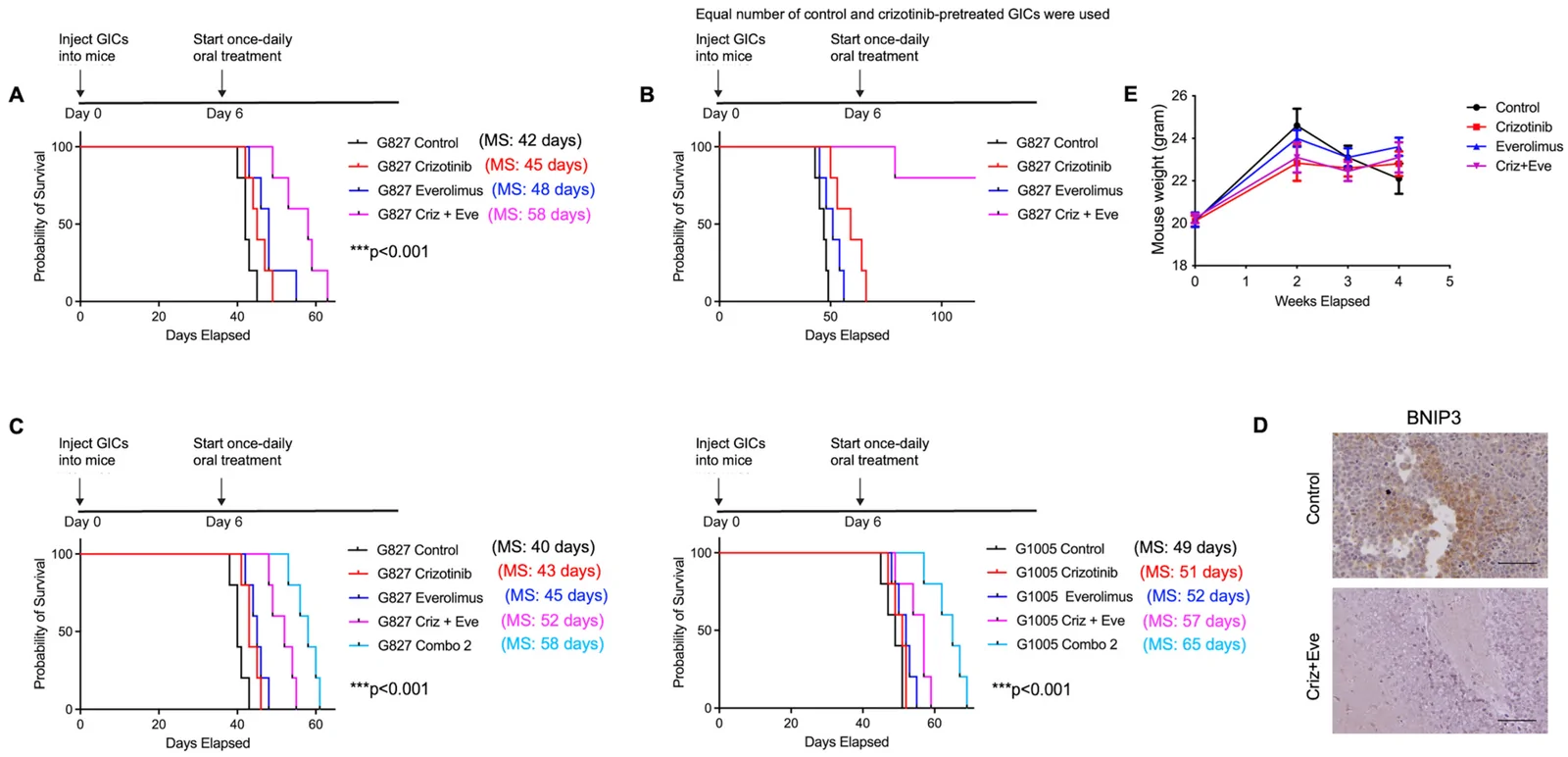
**In vivo efficacy of combined MET and mTOR inhibition.** (**A**,**B**) Kaplan–Meier survival curves of mouse xenografts derived from (**A**) untreated parental GICs and (**B**) crizotinib-pretreated GICs. Mice received continuous daily treatment with crizotinib and everolimus starting at Day 6. (n = 5 mice/group). (**C**) Kaplan–Meier survival curves of mouse xenografts derived from two GIC lines treated with either (i) a continuous daily regimen of crizotinib and everolimus or (ii) a sequential combination regimen (Combo 2), in which crizotinib alone was administered daily for the first 10 days, followed by daily co-treatment of crizotinib and everolimus from day 11 onward (n = 5 mice/group). The sequential treatment group (Combo 2) showed significantly improved survival compared with all other treatment groups, including the concurrent combination group (Combo 1) (*p* < 0.001). (**D**) Immunohistochemical analysis of BNIP3 in orthotopic GBM xenografts from control and combination-treated groups, showing marked downregulation of BNIP3 following Criz+Eve treatment. Scale bar, 50 µm. (**E**) Comparison of mouse body weights across treatment groups.

Next, we evaluated the efficacy of the combined crizotinib and everolimus treatment in orthotopic GBM models. Mice were treated with vehicle, once-daily oral crizotinib (50 mg/kg/day), once-daily oral everolimus (5 mg/kg/day), or the combination of both agents. While single-agent treatments showed minimal therapeutic benefit, combination therapy significantly prolonged both median and overall survival (Figure 6A and Figure S6A). Given the distinct cell death mechanisms observed between untreated and crizotinib-pretreated GICs, we next evaluated the efficacy of the combination therapy in crizotinib-pretreated GICs. After three cycles of in vitro crizotinib treatment, an equal number of parental and crizotinib-pretreated GICs were implanted intracranially to establish xenografts. Daily administration of crizotinib and everolimus markedly extended survival in mice bearing crizotinib-pretreated GIC-derived xenografts, with 4 of 5 mice remaining alive at day 120 (Figure 6B). Building on these findings, we implemented a dosing regimen designed to mimic the in vitro crizotinib treatment cycles, in which crizotinib alone was administered daily for the first 10 days, followed by daily co-treatment with crizotinib and everolimus from day 11 onward (Combo #2). This modified schedule further improved survival compared with the standard combination regimen (Figure 6C). In line with our in vitro findings, immunohistochemical analyses of tumor tissues confirmed suppression of BNIP3 expression and mTOR activity following combination treatment (Figure 6D and Figure S6B). Finally, we monitored mouse body weights across all treatment groups as an indicator of treatment tolerability. As shown in Figure 6E, body weights remained comparable between control and treatment groups, with no significant weight loss observed following either single-agent or combination therapy. Overall, our findings demonstrate that crizotinib and everolimus act synergistically through multiple mechanisms, resulting in significant antitumor efficacy against GICs (Graphical Abstract).

## 3. Discussion

GBM remains one of the most aggressive and treatment-resistant human malignancies, characterized by rapid disease progression and dismal patient survival. Despite aggressive multimodal treatment approaches, tumor recurrence is inevitable, underscoring the urgent need for more effective and durable therapeutic strategies. In this context, our study identifies combined targeting of MET and mTOR signaling as a mechanistically informed approach to overcome adaptive resistance in GBM.

We demonstrate that MET expression is markedly elevated in GBM, particularly in the mesenchymal subtype, which is associated with increased invasiveness, therapeutic resistance, and poor patient outcomes [20,23]. Consistent with these observations, pharmacologic inhibition of MET using crizotinib induces a senescence-like phenotype in GICs, accompanied by suppression of DNA synthesis, cell cycle progression, and proliferative capacity. However, the partial reversibility of this senescence-like state following crizotinib withdrawal indicates that MET inhibition alone is insufficient to induce durable tumor suppression, highlighting the need for rational combination strategies to prevent relapse and adaptive resistance.

A central finding of our study is the identification of a BNIP3-mTOR signaling axis as a critical mediator of resistance to MET inhibition. BNIP3, a HIF1α-regulated mitochondrial protein, plays a key role in mitophagy and cellular adaptation to metabolic and hypoxic stress [24,25]. The downregulation of BNIP3 following MET inhibition suggests a potential mechanism contributing to altered mitochondrial homeostasis and metabolic adaptation, processes that are recognized hallmarks of cancer cell metabolic reprogramming [26,27,28]. Our findings further support a reciprocal regulatory relationship between BNIP3 and mTOR signaling. While BNIP3 depletion activates mTOR signaling, consistent with previous reports demonstrating BNIP3-mediated inhibition of mTOR through Rheb interaction [22], mTOR signaling positively regulates BNIP3 expression. Thus, these findings suggest a negative feedback circuit in which mTOR signaling promotes BNIP3 expression, whereas BNIP3 restrains mTOR activity. We demonstrate that BNIP3 downregulation results in activation of mTOR signaling, thereby promoting tumor cell survival and adaptive resistance. Although combined treatment further reduced BNIP3 expression in vivo, this finding does not contradict the proposed mechanism. BNIP3 downregulation represents an adaptive event that promotes mTOR activation and creates a therapeutic dependency on mTOR signaling. Thus, the efficacy of everolimus does not result from reversing BNIP3 loss, but from blocking the downstream survival pathway activated by BNIP3 suppression. This BNIP3–mTOR regulatory circuit is particularly relevant because mTOR is a master regulator of cellular metabolism and stress adaptation [29,30,31]. Although our findings suggest that BNIP3 downregulation contributes to altered mitochondrial homeostasis during MET inhibition, the direct effects of BNIP3 loss on mitochondrial mass and mitophagy flux were not directly assessed and warrant further investigation. This finding uncovers a key adaptive mechanism driving resistance to MET inhibition, offering a strong rationale for dual targeting of MET and mTOR.

We demonstrate that pharmacologic inhibition of mTOR with everolimus markedly enhances the anti-tumor effects of crizotinib. Combined treatment induces pronounced senescence-associated changes, apoptotic signaling, and alterations in mitochondrial homeostasis, resulting in reduced cell viability, impaired sphere-forming capacity, and significantly prolonged survival in orthotopic GBM xenograft models. These results are consistent with prior studies reporting enhanced therapeutic efficacy of combined MET and mTOR inhibition in other malignancies [32,33,34], and extend these findings by elucidating a mechanistic basis for synergy in GBM.

Notably, our study reveals a context-dependent shift in the mode of cell death following combined MET and mTOR inhibition. While the combined treatment induces robust apoptotic signaling in untreated parental GICs, crizotinib-pretreated cells exhibit a caspase-independent cell death response with molecular features consistent with necroptosis upon subsequent mTOR inhibition. This suggests that prolonged MET inhibition primes tumor cells for alternative stress-induced death pathways. Everolimus-mediated disruption of mTOR signaling likely imposes severe metabolic and survival stress. Under these stress conditions, cells may become less capable of initiating apoptosis and instead engage alternative regulated cell death pathways with necroptotic features. Unlike apoptosis, necroptotic cell death is caspase-independent and can be activated under conditions of overwhelming cellular stress, leading to membrane disruption and inflammatory signaling [35]. This phenomenon may represent a unique therapeutic opportunity, as engagement of necroptotic pathways could potentially overcome the limitations of apoptosis-driven therapies, particularly in tumor types that exhibit resistance to conventional treatments.

In vivo, we further demonstrate that treatment scheduling critically influences therapeutic outcome. While concurrent administration of crizotinib and everolimus significantly prolongs survival, a sequential regimen—initial MET inhibition followed by mTOR blockade—yields superior survival benefit. This enhanced efficacy may reflect progressive activation and increased reliance on mTOR signaling during prolonged MET inhibition, creating an adaptive state of mTOR dependency that sensitizes tumor cells to subsequent mTOR blockade. These findings highlight the importance of therapeutic sequencing in maximizing treatment efficacy and overcoming adaptive resistance in GBM.

Despite these promising findings, several limitations must be considered. The experimental models employed, including GIC cultures and orthotopic xenografts, do not fully recapitulate the complexity of human GBM. In particular, necroptosis is inherently pro-inflammatory and may influence anti-tumor immune responses. Because our models lack an intact immune system, the contribution of immune-mediated mechanisms could not be assessed and should be explored in future studies using immunocompetent systems. Additionally, while our work focuses on MET-mTOR signaling, other compensatory pathways may also contribute to resistance, particularly within the mesenchymal GBM subtype, and merit further investigation.

An important consideration for clinical translation of this therapeutic strategy is drug exposure within the central nervous system (CNS). While everolimus has demonstrated CNS activity and penetration, the extent of crizotinib exposure within GBM tissue remains an important translational consideration. In the current study, we did not directly evaluate plasma or brain drug concentrations, and therefore the relationship between drug exposure and the therapeutic effects observed with combination treatment remains unknown. Future pharmacokinetic studies will be important to evaluate drug exposure in GBM. Another limitation of this study is that all in vivo experiments were performed using female mice. Although this design allowed consistency across experimental cohorts, sex-dependent differences in GBM biology and therapeutic responses have been reported. Future studies incorporating both male and female models will be important to determine whether the therapeutic effects of MET and mTOR co-inhibition are influenced by sex.

In summary, our study identifies a previously unrecognized BNIP3–mTOR signaling axis as a key determinant of resistance to MET inhibition in GBM. Dual targeting of MET and mTOR overcomes adaptive resistance, engages multiple cell death pathways, and significantly improves therapeutic efficacy in preclinical models without overt toxicity. These findings provide a strong mechanistic and translational rationale for clinical evaluation of combined MET and mTOR inhibition in patients with GBM, particularly those with high MET expression.

## 4. Materials and Methods

### 4.1. Cell Culture, Cell Viability Detection, Self-Renewal Assays, and Reagents

Primary glioma-initiating cell (GIC) lines were obtained from University of Alabama and Jeongwu Lee (Cleveland Clinic) and have been published previously [36,37,38]. The human identity of all cell lines was verified by short tandem repeat (STR) profiling prior to experimentation. GIC identity was further confirmed by the expression of established stem cell markers, including SOX2, CD133, OLIG2, and CD44. All cell lines were routinely tested for mycoplasma contamination by PCR, with testing repeated every five weeks. To minimize genetic drift and ensure experimental reproducibility, all experiments were performed using low-passage cells (<10 passages). Cultures were periodically reinitiated from early-passage stocks every four weeks. GICs were maintained as floating neurospheres in Neurobasal medium supplemented with glutamine, N2 (ThermoFisher Scientific, Waltham, MA, USA), B27 (ThermoFisher Scientific), epidermal growth factor (EGF; 20 ng/mL; R&D Systems, Minneapolis, MN, USA), and fibroblast growth factor (FGF; 20 ng/mL; R&D Systems) at 37 °C in a humidified incubator with 5% CO_2_. For in vitro assays, neurospheres were dissociated into single cells using 0.02% ethylenediaminetetraacetic acid (EDTA; Lonza, Basel, Swiss) and plated onto laminin-coated culture plates (Corning, Corning, NY, USA). Self-renewal assays were conducted using ultra-low attachment plates (Corning). Neurosphere formation was quantified following drug treatment, and analyses were performed as previously described [39]. Cell viability was assessed 72 h after treatment using both the CellTiter-Glo luminescent assay (Promega, Madison, WI, USA) and Trypan Blue exclusion with automated cell counting (Cellometer Auto T4; Nexcelom, Lawrence, MA, USA). Crizotinib (HY-50878) was purchased from MedChemExpress, Monmouth Junction, NJ, USA and everolimus (S1120) was obtained from Selleckchem, Houston, TX, USA.

### 4.2. Animal Studies

All animal studies were approved by the Institutional Animal Care and Use Committee (IACUC) at Penn State University and conducted in accordance with institutional guidelines. Six-to-eight-week-old female BALB/c SCID NCr mice were used for all in vivo experiments. A total of 150,000 G827 or G559 GICs were stereotactically injected into the right striatum of each mouse. Following tumor implantation, mice were randomized into four treatment groups: vehicle control, crizotinib alone, everolimus alone, or combination treatment. Two treatment regimens were employed: (i) a continuous treatment regimen, in which crizotinib and everolimus were administered daily throughout the study and (ii) a sequential combination regimen (Combo 2), in which crizotinib alone was administered daily for the first 10 days, followed by daily co-treatment with crizotinib and everolimus from day 11 onward. Both drugs were administered once daily by oral gavage. Crizotinib and everolimus were dosed at 50 mg/kg and 5 mg/kg, respectively, based on our experience and published studies [15,38]. Mice were monitored daily for neurological symptoms and overall health and were euthanized upon reaching predefined humane endpoints or terminal disease. Overall survival was recorded and compared between treatment groups. No animals were excluded from the survival analysis. Sample size for each group was determined based on prior experience and power calculations, assuming a median survival of 40 days for the control group and 60 days for treated groups, and a statistical power of 85%.

### 4.3. Immunoblotting, Caspase-3/7 Assay, and qPCR

Immunoblotting was performed as previously described [40]. The following primary antibodies were used for immunoblot: β-actin (A5441) and GAPDH (G9545) were obtained from Sigma-Aldrich, Saint Louis, MO, USA; phospho-mTOR (Ser2448; #2971), phospho-p70 S6 kinase (Thr389; #9234), phospho-S6 (Ser235/236; #4858), phospho-RIP1 (#65746), BNIP3 (#44060), and H3K27me3 (#9733) were obtained from Cell Signaling Technology, Danvers, MA, USA; H3K9me2 (ab1220) was obtained from Abcam, Waltham, MA, USA; histone H3 (05-499) was obtained from MilliporeSigma, Saint Louis, MO, USA; and phospho-MLKL (PA5-105678) and cleaved caspase-9 (PA5-105271) were obtained from Thermo Fisher Scientific, Waltham, MA, USA. Caspase-3/7 activity was measured using the Caspase-Glo^®^ 3/7 Assay kit (G8090; Promega) according to the manufacturer’s instructions following 48 h of drug treatment. Total RNA was isolated using QIAzol reagent (Qiagen, Germantown, MD, USA) and reverse-transcribed into cDNA using the SuperScript™ III First-Strand Synthesis Kit (Invitrogen, Waltham, MA, USA), following the manufacturer’s instructions. Quantitative real-time PCR was performed using 2 µL of diluted cDNA on an Applied Biosystems StepOnePlus PCR system with Power SYBR™ Green PCR Master Mix (Applied Biosystems, Foster City, CA, USA). Relative gene expression levels were calculated for each sample and normalized to *GAPDH* expression for comparative analysis.

The following primer sequences were used for qPCR (5′ → 3′):

- *IL6*: F: ACTCACCTCTTCAGAACGAATTG, R: CCATCTTTGGAAGGTTCAGGTTG.
- *CXCL10*: F: GTGGCATTCAAGGAGTACCTC, R: TGATGGCCTTCGATTCTGGATT.
- *CXCL8*: F: TTTTGCCAAGGAGTGCTAAAGA, R: AACCCTCTGCACCCAGTTTTC.
- *BNIP3*: F: CAGGGCTCCTGGGTAGAACT, R: CTACTCCGTCCAGACTCATGC.

### 4.4. Plasmid and siRNA Transfection

An mTOR expression plasmid (Addgene #69010), along with the corresponding control plasmid, was used for rescue experiments. A total of 125,000 cells were seeded onto laminin-coated 12-well plates, and plasmid transfection was performed the following day using FuGENE^®^ HD transfection reagent (Promega) according to the manufacturer’s instructions. Twenty-four hours after transfection, cells were treated with a combination of crizotinib and everolimus. Cell numbers were quantified and compared after 48 h of treatment. Non-targeting control siRNA and siRNAs targeting *c-MET* and *BNIP3* were obtained from Dharmacon, Lafayette, CO, USA (SMARTpool ON-TARGETplus). siRNA transfections in GICs were performed using Lipofectamine^®^ RNAiMAX (Thermo Fisher Scientific) in accordance with the manufacturer’s protocol, with a final siRNA concentration of 10 nmol/L.

### 4.5. Bulk RNA-Seq and Bioinformatic Analysis

For the three cell lines we used in this study, we used two technical replicates for RNA-Seq experiments and analyses for each individual condition. For each replicate, total RNA was extracted using the RNeasy Kit (Qiagen), and sequencing libraries were constructed with the NEB Next Ultra RNA Library Prep Kit for Illumina platform sequencing. Paired-end RNA sequencing reads were aligned to the human reference genome (hg38) using the STAR aligner [41], and uniquely mapped reads were retained for downstream analysis. Gene-level quantification was performed using Cufflinks [42] with the Gencode v38 annotation [43], and expression levels were reported as fragments per kilobase of transcript per million mapped reads (FPKM). Genes with an FPKM greater than 1.0 in at least two samples from either experimental group were retained for downstream analysis. Raw read counts were generated using the *featureCounts* function from the Rsubread package (version 2.18.0) [44]. Differential expression analysis was carried out using edgeR [45]. Raw counts were normalized using the trimmed mean of M-values (TMM) method, and a quasi-likelihood negative binomial generalized log-linear model was fit using the glmQLFit and glmQLFTest functions. Genes with a false discovery rate (FDR) at or below 0.05 and fold change ≥ 1.5 were considered significantly differentially expressed. Gene set enrichment analysis (GSEA) was performed using the GSEA software suite (version 4.3.3) [46] with normalized FPKM values as input. Prior to analysis, genes with zero expression in either comparison group were removed, and any infinite values produced by log2 class ratio calculations were replaced with finite values slightly beyond the observed maximum or minimum for that gene, preserving rank order while avoiding undefined scores. Visualization of differential expression and enrichment results was performed using R packages including *ggplot2* (*version 3.5.2*), *pheatmap* (*version 1.0.12*), *enrichplot* (*version 1.26.6*), and *dplyr* (*version 1.1.4*).

### 4.6. Mitochondrial Membrane Potential Analysis (JC-1 Staining)

Mitochondrial membrane potential was assessed using JC-1 dye (Thermo Fisher Scientific) according to the manufacturer’s instructions. Briefly, GICs were seeded onto laminin-coated plates and treated as indicated. Following treatment, cells were incubated with JC-1 working solution at 37 °C for 20 min in the dark. After incubation, cells were washed twice with assay buffer and analyzed immediately by fluorescence microscopy. JC-1 aggregates (red fluorescence) indicate healthy mitochondria with high membrane potential, whereas JC-1 monomers (green fluorescence) indicate depolarized mitochondria.

### 4.7. Mitochondrial Stress Test

Mitochondrial Stress Test was performed as described previously [38]. Cells were seeded as a monolayer onto a laminin-coated Seahorse 24-well tissue culture plate (Agilent Technologies, Santa Clara, CA, USA). After 48 h of drug treatment, the media were replaced with DMEM supplemented with pyruvate, and the cells were allowed to equilibrate for 30 min at 37 °C in a CO_2_-free incubator. Oxygen consumption rate (OCR) was measured using a Seahorse XF24 Flux Analyzer (Agilent Technologies, Santa Clara, CA, USA). After three baseline measurements, compounds were sequentially injected: 1 µM Oligomycin (Sigma-Aldrich), 2 µM BAM15 (Cayman Chemical, Ann Arbor, MI, USA), and 1 µM Antimycin A & 100 nM Rotenone (Sigma-Aldrich). Basal respiration was calculated by subtracting the average OCR after Antimycin A & Rotenone treatment from the first three baseline measurements. Maximum respiratory capacity was determined by subtracting the post-BAM15 OCR from the post-Antimycin A & Rotenone OCR. Reserve capacity was calculated by subtracting the basal OCR from the post-BAM15 OCR.

### 4.8. β-Galactosidase and Immunohistochemistry (IHC) Staining

Senescence-associated β-galactosidase (SA-β-Gal) activity was assessed using a β-Gal staining kit (Abcam) according to the manufacturer’s instructions. Briefly, GICs were seeded onto laminin-coated plates and treated as indicated. After treatment, cells were washed with PBS and fixed using the provided fixation solution for 10–15 min at room temperature. Following fixation, cells were incubated with β-Gal staining solution at 37 °C (without CO_2_) overnight. Blue-stained cells were visualized and quantified using bright-field microscopy. IHC staining was performed as described previously [47]. Tissue slides were fixed in a 1:1 mixture of acetone and methanol for 10 min at room temperature. Endogenous peroxidase activity was quenched using peroxidase inhibitor (CM1; Ventana, Tucson, AZ, USA) for 8 min. Slides were then incubated with primary antibodies at room temperature. Antigen–antibody complexes were subsequently detected using the DISCOVERY OmniMap Anti-Rabbit HRP detection system in combination with the DISCOVERY ChromoMap DAB Kit (Ventana Medical Systems), following the manufacturer’s instructions. Stained slides were visualized by bright-field microscopy.

### 4.9. Statistics and Synergy Calculations

All statistical analyses were performed using GraphPad Prism 10 (GraphPad Software). For comparisons between two groups, Student’s *t*-test was applied, while one-way ANOVA followed by Tukey’s post-hoc test was used for multiple group comparisons. *p*-values < 0.05 were considered statistically significant (α = 0.05). Kaplan–Meier survival analysis was used to generate mouse survival curves. Survival differences between treatment groups were assessed using the Mantel–Cox (log-rank) test. Pairwise comparisons were performed with Bonferroni correction for multiple comparisons. Synergy between treatments was evaluated using two independent methods: the Bliss independence model and the Chou–Talalay method (ComboSyn). Combination indices (CI) were calculated according to the Chou–Talalay method, with CI < 1 indicating synergy and CI < 0.1 indicating very strong synergy [48]. The Bliss difference was calculated as described previously [49]. Briefly, the Bliss value was obtained by subtracting the predicted cytotoxicity, based on individual treatments, from the observed cytotoxicity of the combination therapy. A Bliss value of zero corresponds to an additive effect, a value greater than zero indicates synergy, and a value less than zero indicates antagonism. This approach provides informative synergy assessment even when one component of a combination exhibits minimal effect.

### 4.10. Data Availability

RNA-sequencing data generated during this study are available in the GEO repository under accession number GSE320374. The raw images and datasets are available on request from the corresponding authors upon request.

### 4.11. Code Availability

No code was generated/used in the preparation of this paper.

## Data Availability

The original contributions presented in this study are included in the article/Supplementary Material. Further inquiries can be directed to the corresponding author.

## Supplementary Materials

The following supporting information can be downloaded at: https://www.mdpi.com/article/10.3390/ijms27177780/s1.
