# Supplementary material for: Targeting MET and mTOR Synergistically Overcomes Adaptive Resistance in Glioblastoma
Source: Int J Mol Sci. 2026 Aug 30;27(17):7780. doi: 10.3390/ijms27177780 (PMC13565985; doi:10.3390/ijms27177780)
Supplement: Supplementary file 1 [file ijms-27-07780-s001.zip › ijms-4483997-supplementary.pdf]

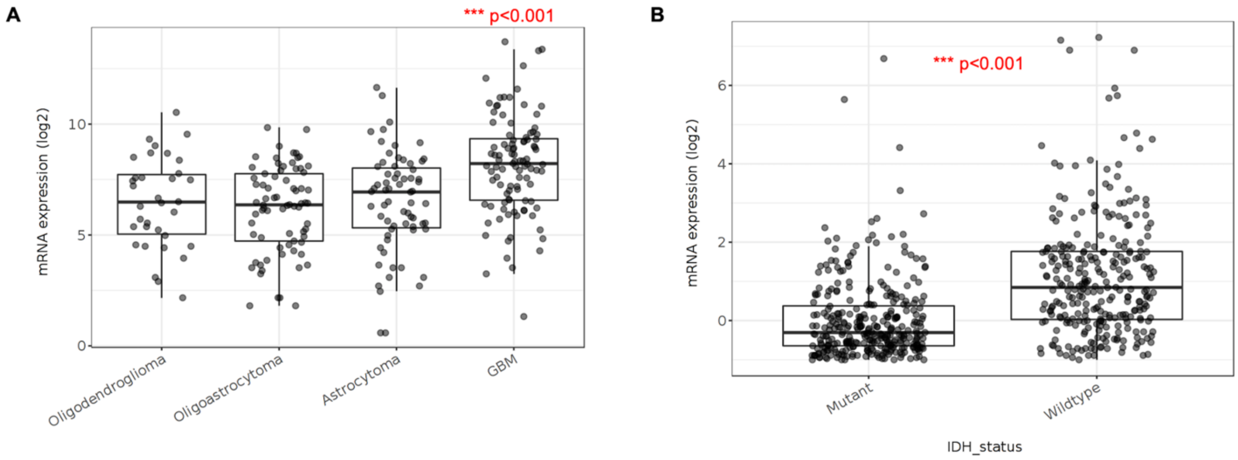

Supplementary Figure S1: High MET expression is associated with glioblastoma and higher glioma grade. (**A**) MET mRNA expression across glioma subtypes in the TCGA dataset, including oligodendroglioma, oligoastrocytoma, astrocytoma, and glioblastoma (GBM), showing significantly higher MET expression in GBM compared with other glioma subtypes. (**B**) TCGA analysis of MET mRNA expression in GBM according to IDH status, demonstrating elevated MET expression in IDH wild-type compared with IDH-mutant tumors.

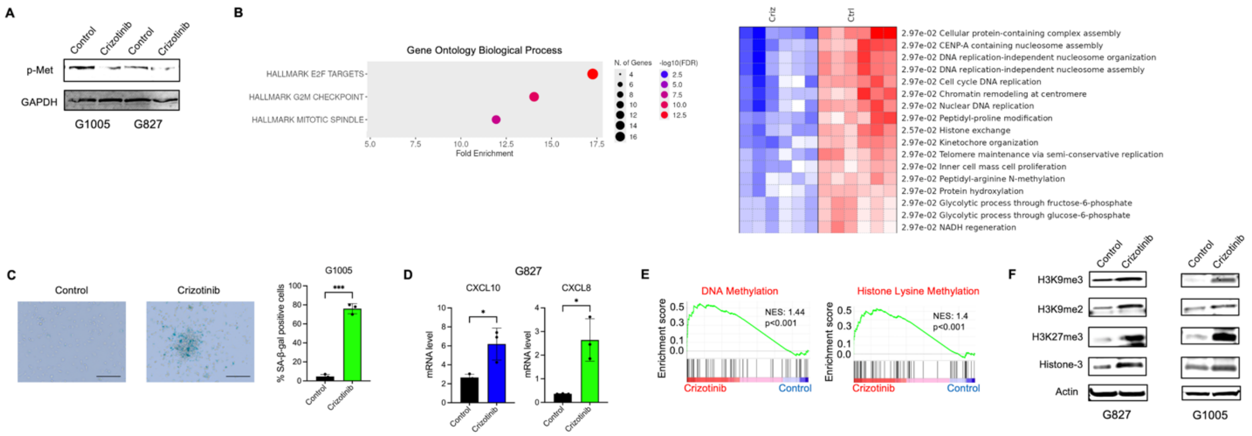

Supplementary Figure S 2: Crizotinib treatment induces transcriptional, epigenetic, and senescence-associated changes in GICs. (A) Immunoblot showing decreased Met activity following crizotinib treatment. (B) Gene Ontology (Biological Process) and Hallmark pathway enrichment analysis of RNA-seq data. (C) Representative images of senescence-associated β-galactosidase (SA-β-gal) staining in control and crizotinib-treated GICs, showing increased senescent cells upon MET inhibition. Crizotinib (0.75 µM). Scale bar, 200 µm. Data are shown as mean ± SEM. (****p < 0.0001; two-tailed *t*-test). (D) Quantitative RT–PCR analysis of CXCL10 and CXCL8 mRNA expression in GICs following crizotinib (0.75 µM) treatment. Data represent mean ± SEM. $P < 0.05$ by two-tailed *t*-test. (E) GSEA showing enrichment of DNA methylation– and histone lysine methylation–related gene sets in crizotinib-treated cells compared with control cells. (F) Immunoblot analysis of histone methylation marks in G827 and G1005 cells treated with vehicle or crizotinib.

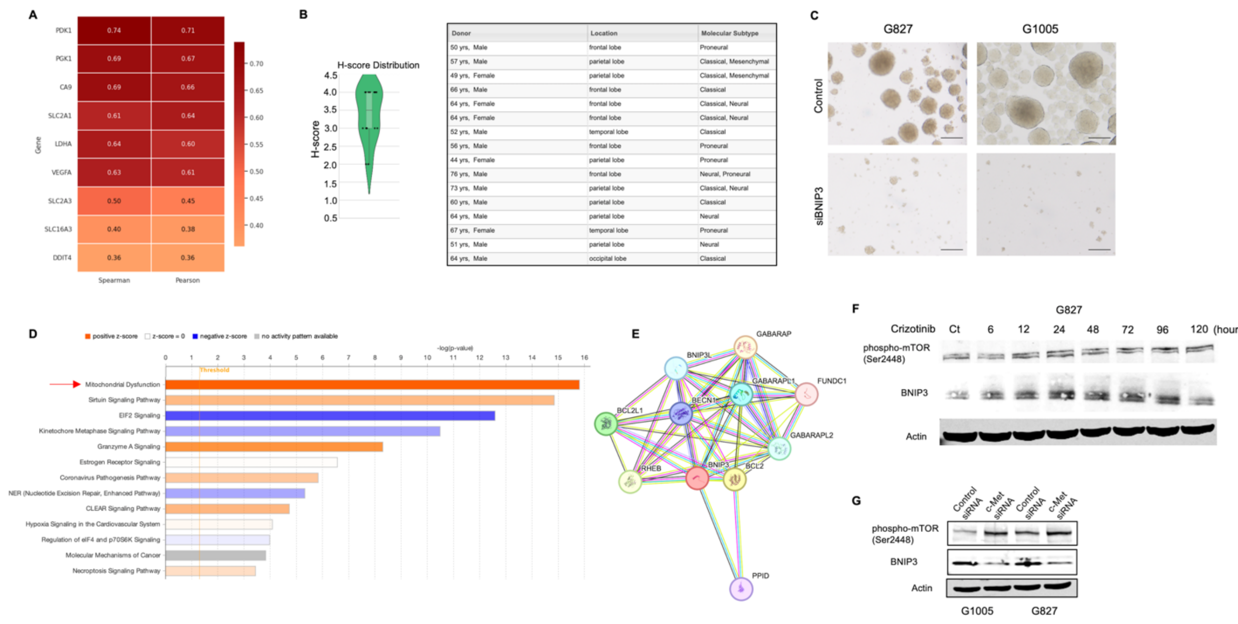

Donor	Location	Molecular Subtype
50 yrs, Male	frontal lobe	Proneural
57 yrs, Male	parietal lobe	Classical, Mesenchymal
49 yrs, Female	parietal lobe	Classical, Mesenchymal
66 yrs, Male	frontal lobe	Classical
64 yrs, Female	frontal lobe	Classical, Neural
64 yrs, Female	frontal lobe	Classical, Neural
52 yrs, Male	temporal lobe	Classical
56 yrs, Male	frontal lobe	Proneural
44 yrs, Female	parietal lobe	Proneural
76 yrs, Male	frontal lobe	Neural, Proneural
73 yrs, Male	parietal lobe	Classical, Neural
60 yrs, Male	parietal lobe	Classical
64 yrs, Male	parietal lobe	Neural
67 yrs, Female	temporal lobe	Proneural
51 yrs, Male	parietal lobe	Neural
64 yrs, Male	occipital lobe	Classical

Supplementary Figure S3: BNIP3 links mitochondrial dysfunction and hypoxia signaling to tumor spheroid growth. (A) Heatmap showing Spearman and Pearson correlation coefficients between BNIP3 expression and selected HIF1alpha-related genes. (B) Quantification of BNIP3 protein expression using H-score analysis of human GBM samples. All samples included in this analysis were IDH-wildtype. n = 16 (Ivy GBM Atlas Project). (C) 100 GICs were cultured in 24-well plates over one week to assess self-renewal capacity upon transfection with control siRNA or BNIP3-targeting siRNA. Scale bars, 100 µm. (D) Ingenuity Pathway Analysis of differentially expressed genes, highlighting significantly enriched canonical pathways. (E) Protein–protein interaction network of BNIP3. (F) Time-course immunoblot analysis of GICs treated with crizotinib (0.75 µM) at the indicated time points, demonstrating progressive mTOR activation concomitant with BNIP3 downregulation during prolonged MET inhibition. (G) Immunoblot analysis of phospho-mTOR (Ser2448) and BNIP3 levels in GICs transfected with control siRNA or MET-targeting siRNA. Actin was used as a loading control.

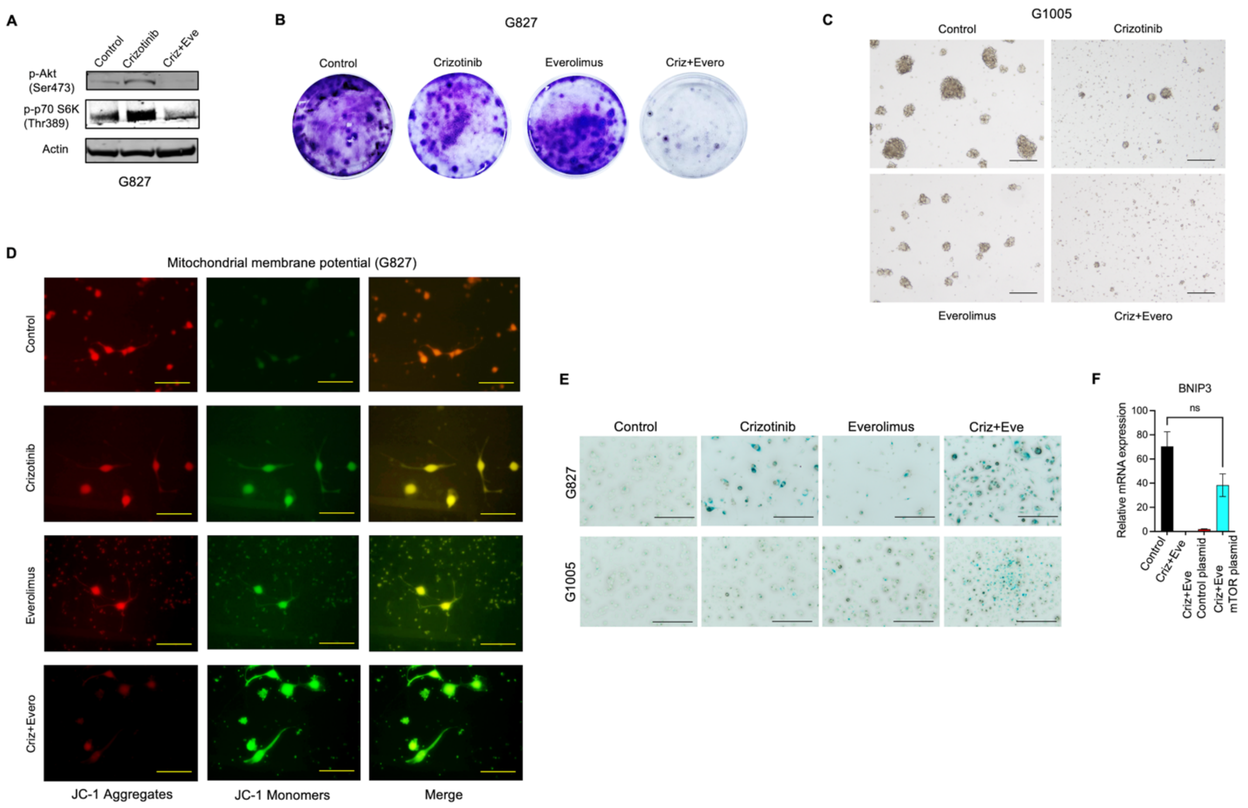

Supplementary Figure S4: Combined inhibition of MET and mTOR signaling modulates tumor cell growth and mitochondrial function. (A) Immunoblot analysis in GICs treated with control, crizotinib, or their combination (Criz+Evero). (B) Representative colony formation assay images of G827 cells under indicated treatments. (C) 100 GICs were cultured in 24-well plates over one week to assess self-renewal capacity under vehicle, crizotinib (0.75 µM), everolimus (5 µM), or combination treatment. Scale bars, 100 µm. (D) Assessment of mitochondrial membrane potential in G827 cells using JC-1 staining. Red fluorescence indicates JC-1 aggregates (intact mitochondrial membrane potential), whereas green fluorescence indicates JC-1 monomers (depolarized mitochondria). Scale bars, 200 µm. (E) Representative images of senescence-associated β-galactosidase staining. Scale bar, 200 µm. (F) Quantitative RT–PCR analysis of BNIP3 mRNA expression under indicated treatments. Data are mean ± SEM (n = 3). One-way ANOVA with Tukey's post hoc test; ns = not significant.

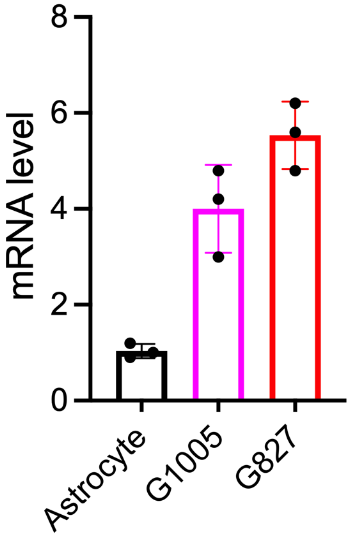

Supplementary Figure S5: RIPK3 expression is increased in GSCs. Quantitative RT-PCR analysis of RIPK3 mRNA expression in GSC lines and astrocytes.

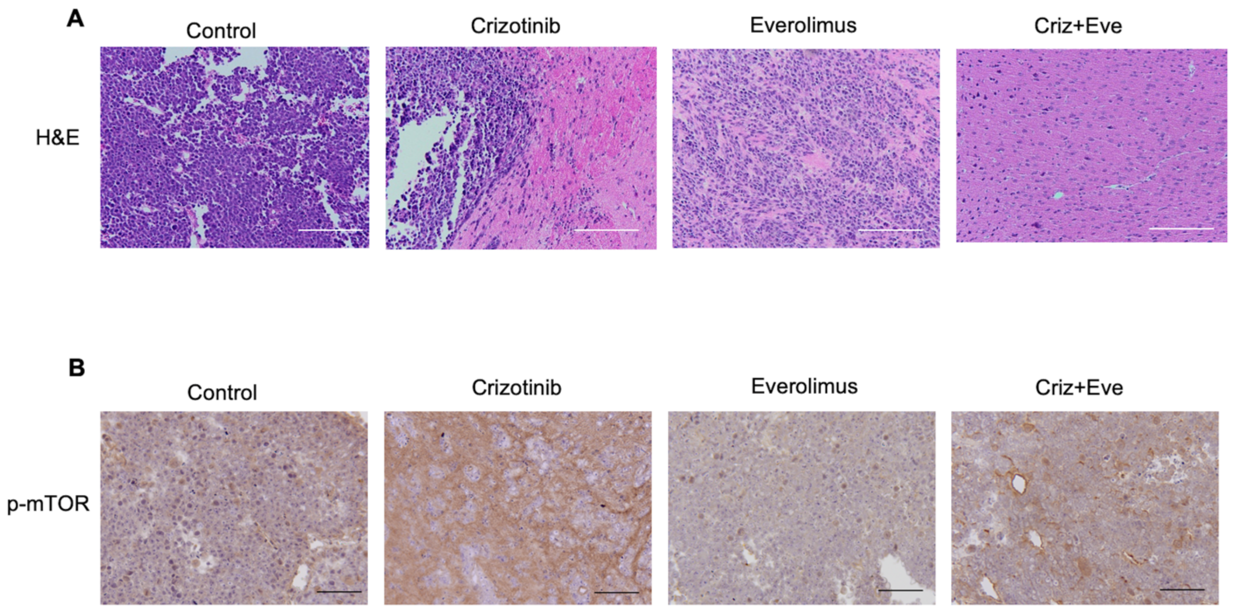

Supplementary Figure S6: Combined crizotinib and everolimus suppresses tumor growth and mTOR signaling. (A) Representative H&E staining of orthotopic GBM xenografts from control, crizotinib-, everolimus-, and combination-treated groups, showing treatment-associated changes in tumor morphology. Scale bar, 100 μm (B) Immunohistochemical staining of orthotopic GBM mouse xenografts from control, crizotinib, everolimus, or Criz+Eve groups, demonstrating suppression of mTOR signaling upon combination treatment. Scale bar, 50 μm.

Supplementary Table S1: Genes significantly downregulated following crizotinib treatment

**Gene Name**
EDIL3
VSNL1
CLDN11
HAPLN1
**MYCBP**
LDHAP5
P4HA1
RP11-344N10.2
LDHA
LDHAP7
HIST1H4C
LDHAP4
PGK1P2
MAD2L1
PMP2
HMGB1P10
HIST1H1E
MTND4P12
HMGB1P5
LDHAP2
RP11-673C5.1
HMGB1
ACADM
SLC2A3
HILPDA
MT-ND4
MT-ND4L
MT-CO3
RP11-192H23.5
CSRP2
HMGB2
PTTG1
PTN
TMSB15A
PIGA
FABP7
CDK1

DLGAP5
TTK
KIF18A
ARSK
EPHA3
RP11-
287D1.3
MT-ND5
EIF2A
ANLN
SLC37A4
PGK1
DYNLT3
DHFRP1
DEPDC1B
FKBP7
TPI1P1
COMMD10
PBK
RP11-
802O23.3
PPIAP31
FAM162A
BNIP3
RABL3
SUCLG2
CENPE
TPI1
MT-ND6
CYCS
FKBP3
NUDCD1
XRCC4
MT-CYB
MTHFD2
PSPHP1
OSTF1
PDHB
RAD51AP1
UQCRC2
MZT1
RP11-
15B17.1

NUP107
IGFBP2
PAICS
CLDND1
CENPW
HSP90B1
BUB1
PGM3
DPCD
THOC3
BHLHE40
NUF2
SLC4A7
PON2
ITGB3BP
CCNA2
OSTC
TOP2A
RRM1
EMC2
NDUFS4
UBE2T
KNSTRN
EXOSC9
BORA
GGH
RRM1-AS1
RP11-77H9.2
GALK2
SAAL1
RPE
NUSAP1
PRPS1
SSR3
DDX10
MCTS1
SMC2
ECT2
PCCA
ACTL6A
ZWINT
CEP55
ARL1

ERO1L
MTATP6P1
NUP37
FAM64A
PRPF39
CARHSP1
RP11-
196G11.3
PGAM1
EXOSC8
CCNB2
CTB-167B5.1
STT3B
SRI
WDR54
KIF2C
